# Complete mitochondrial genome of *Pseudophlugiolopsis bitubera* (Orthoptera:Tettigoniidae:Meconematinae)

**DOI:** 10.1128/mra.01112-25

**Published:** 2026-04-29

**Authors:** Zhihong Zhang, Tingting Yu, Xun Bian, Bin Zhang

**Affiliations:** 1 College of Life Sciences & Technology, Inner Mongolia Normal University 71203 , Hohhot, China; 2 Key Laboratory of Ecology of Rare and Endangered Species and Environmental Protection (Guangxi Normal University), Ministry of Education 12388, Guilin, China; 3 Key Laboratory of Biodiversity conservation and Sustainable utilization for College and University of Inner Mongolia Autonomous Region, Hohhot, China; University of California Riverside, Riverside, California, USA

**Keywords:** mitogenome, China, *Pseudophlugiolopsis*, Meconematinae, Orthoptera

## Abstract

We report the whole mitochondrial genome of the Chinese species *Pseudophlugiolopsis bitubera*. The mitogenome of *P. bitubera* is circular, AT-rich (70.3%), and 15,611 bp in length. It comprises 13 protein-coding genes, two ribosomal RNA genes, and 22 transfer RNA genes.

## ANNOUNCEMENT

The genus *Pseudophlugiolopsis* (monotypic with *P. bitubera* (1) originally described from specimens from Xinjie, Yuanyang, Yunnan, China) is similar to the genus *Phlugiolopsis* Zeuner, 1940; however, it is distinguished by the posterior margin prolongation of the male 10th abdominal tergite bearing a pair of long processes (1). To enrich molecular data and contribute to the phylogeny of *Pseudophlugiolopsis*, the complete mitochondrial genome of *P. bitubera* was assembled and annotated.

The specimen of *P. bitubera* analyzed here was collected from type locality Xinjie, Yuanyang, Yunnan, China (23.1084N 102.7354E). The voucher specimen was stored in absolute ethyl alcohol at −4°C in the College of Life Sciences, Guangxi Normal University (GXNU). Total genomic DNA was extracted from the muscle tissues of the hind leg using the TIANamp Genomic DNA Kit (TIANGEN) following the instructions and sent to Beijing Berry Genomics Co., Ltd. for high-throughput sequencing. The paired-end library was constructed with the MGIEasy Kit (MGI) and sequenced as 2 × 150 bp reads on an Illumina NovaSeq 6000 (Illumina, Inc., San Diego, CA, USA) to produce 12,215,928 read pairs. The raw data were processed with fastp v.0.20.0 by trimming adapters and primers, filtering reads with phred quality < Q5, and filtering reads with *N* base number > 3. After QC filtering, 82,784 read pairs remained for data processing. *Paraphlugiolopsis.jiangi* (NC_068778) was selected as the reference sequence after searching the near-source species in National Center for Biotechnology Information (NCBI). The newly sequenced genome was assembled and processed using NOVOplasty 4.2.1 (2) based on the reference sequence. A single *P. bitubera* mitochondrial contig with 1,565× coverage was identified by Quast 5.2.0 using the default settings. The annotation was performed on the MITOS web server (3). The annotated data were verified using MEGA V.12.1, yielding standardized mitochondrial genome data for *P. bitubera* (4). Adenine-thymine (AT) content, AT skew, and guanine-cytosine (GC) skew were calculated using PhyloSuite v1.2.3 (5). A circular mitogenome map was generated using the Chloroplot server online tool (6).

**Fig 1:**
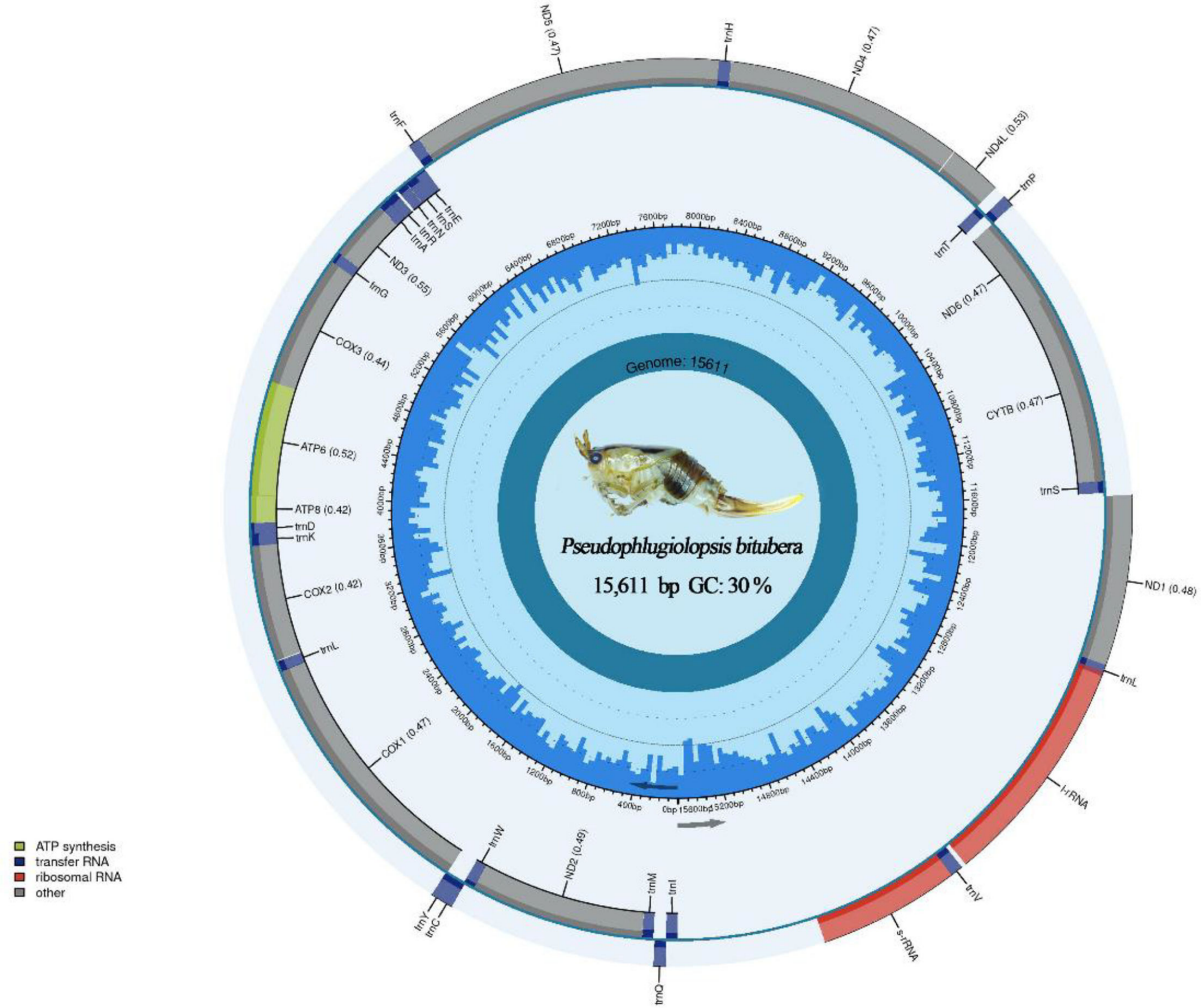
The circle map indicates this specimen’s total genome length and GC content. The innermost ring is labeled with GC content and transcription direction; the outermost ring shows the distribution of genes.

The complete circular mitochondrial genome of *P. bitubera* is 15,611 bp in length and has an AT bias of 70.3% (PX_423276). The GC content is 29.7%. The complete mitogenome contains 37 genes, including 13 protein-coding genes, two ribosomal RNA genes, 22 transfer RNA genes, and a control region (D-loop) (Fig. 1). Except for ND1, which starts with TTG, the other protein-coding genes initiate with ATN (Table 1). The TAA termination codon is found in all genes except ND1 and CYTB (TAG), COX2, COX3, ND5, and ND4 (T, stop codon is completed by the addition of 3′ A residues to the mRNA, as is common in animal mitochondrial genomes (7, 8). The entire mitochondrial genome sequence of *P. bitubera* is 92.14% similar to the genome of *Paraphlugiolopsis jiangi* from China.

**TABLE 1:** Gene annotation and genome structure analysis of *Pseudophlugiolopsis bitubera*

Gene	Type	Minimum nucleotide position	Maximum nucleotide position	Length	Start codons	Stop codons	Direction
tRNA-Ile	tRNA	1	67	67	–^a^	–	Forward
tRNA-Gln	tRNA	64	133	70	–	–	Reverse
tRNA-Met	tRNA	142	207	66	–	–	Forward
nad2	CDS	208	1236	1029	ATT	TAA	Forward
tRNA-Trp	tRNA	1234	1300	67	–	–	Forward
tRNA-Cys	tRNA	1292	1356	65	–	–	Reverse
tRNA-Tyr	tRNA	1356	1420	65	–	–	Reverse
cox1	CDS	1413	2957	1545	ATT	TAA	Forward
tRNA-Leu	tRNA	2952	3017	66	–	–	Forward
cox2	CDS	3023	3707	685	ATG	T	Forward
tRNA-Lys	tRNA	3707	3777	71	–	–	Forward
tRNA-Asp	tRNA	3776	3842	67	–	–	Forward
atp8	CDS	3843	4007	165	ATT	TAA	Forward
atp6	CDS	4001	4678	678	ATG	TAA	Forward
cox3	CDS	4678	5464	787	ATG	T	Forward
tRNA-Gly	tRNA	5464	5529	66	–	–	Forward
nad3	CDS	5530	5883	354	ATT	TAA	Forward
tRNA-Ala	tRNA	5885	5949	65	–	–	Forward
tRNA-Arg	tRNA	5948	6011	64	–	–	Forward
tRNA-Asn	tRNA	6027	6093	67	–	–	Forward
tRNA-Ser	tRNA	6095	6162	68	–	–	Forward
tRNA-Glu	tRNA	6162	6229	68	–	–	Forward
tRNA-Phe	tRNA	6235	6301	67	–	–	Reverse
nad5	CDS	6302	8033	1732	ATT	T	Reverse
tRNA-His	tRNA	8033	8100	68	–	–	Reverse
nad4	CDS	8101	9427	1327	ATT	T	Reverse
nad4l	CDS	9433	9729	297	ATG	TAA	Reverse
tRNA-Thr	tRNA	9731	9798	68	–	–	Forward
tRNA-Pro	tRNA	9797	9862	66	–	–	Reverse
nad6	CDS	9864	10391	528	ATA	TAA	Forward
cob	CDS	10391	11527	1137	ATG	TAG	Forward
tRNA-Ser	tRNA	11525	11594	70	–	–	Forward
nad1	CDS	11612	12559	948	TTG	TAG	Reverse
tRNA-Leu	tRNA	12559	12623	65	–	–	Reverse
16sRNA	rRNA	12598	13907	1310	–	–	Reverse
tRNA-Val	tRNA	13933	14004	72	–	–	Reverse
12sRNA	rRNA	14003	14790	788	–	–	Reverse

^a^ –, not applicable.

## Data Availability

The complete mitochondrial genome sequence of *Pseudophlugiolopsis bitubera* is available in GenBank under accession number PX_423276 The associated BioProject, SRA, and BioSample numbers are PRJNA1335134, SRR35993925, and SAMN51886826, respectively. The mitochondrial genome referenced in the text is *Paraphlugiolopsis jiangi* GenBank accession number NC_068778.
